# A pilot randomised controlled trial of a Telehealth intervention in patients with chronic obstructive pulmonary disease: challenges of clinician-led data collection

**DOI:** 10.1186/1745-6215-15-313

**Published:** 2014-08-06

**Authors:** Claire L Bentley, Gail A Mountain, Jill Thompson, Deborah A Fitzsimmons, Kinga Lowrie, Stuart G Parker, Mark S Hawley

**Affiliations:** School of Health and Related Research, The University of Sheffield, Regent Court, 30 Regent Street, Sheffield, S1 4DA England; School of Nursing and Midwifery, The University of Sheffield, Barber House Annexe, 3a Clarkehouse Road, Sheffield, S10 2LA England; School of Health Studies, Western University, London, ON N6A 3B4 Canada; Institute for Ageing and Health, Newcastle University, Campus for Ageing and Vitality, Newcastle upon, Tyne NE4 5PL UK

**Keywords:** Chronic obstructive pulmonary disease, Implementation, Pilot RCT, Telehealth, Telemonitoring

## Abstract

**Background:**

The increasing prevalence and associated cost of treating chronic obstructive pulmonary disease (COPD) is unsustainable, and focus is needed on self-management and prevention of hospital admissions. Telehealth monitoring of patients’ vital signs allows clinicians to prioritise their workload and enables patients to take more responsibility for their health. This paper reports the results of a pilot randomised controlled trial (RCT) of Telehealth-supported care within a community-based COPD supported-discharge service.

**Methods:**

A two-arm pragmatic pilot RCT was conducted comparing the standard service with a Telehealth-supported service and assessed the potential for progressing into a full RCT. The co-primary outcome measures were the proportion of COPD patients readmitted to hospital and changes in patients’ self-reported quality of life. The objectives were to assess the suitability of the methodology, produce a sample size calculation for a full RCT, and to give an indication of cost-effectiveness for both pathways.

**Results:**

Sixty three participants were recruited (n = 31 Standard; n = 32 Telehealth); 15 participants were excluded from analysis due to inadequate data completion or withdrawal from the Telehealth arm. Recruitment was slow with significant gaps in data collection, due predominantly to an unanticipated 60% reduction of staff capacity within the clinical team. The sample size calculation was guided by estimates of clinically important effects and COPD readmission rates derived from the literature. Descriptive analyses showed that the standard service group had a lower proportion of patients with hospital readmissions and a greater increase in self-reported quality of life compared to the Telehealth-supported group. Telehealth was cost-effective only if hospital admissions data were excluded.

**Conclusions:**

Slow recruitment rates and service reconfigurations prevented progression to a full RCT. Although there are advantages to conducting an RCT with data collection conducted by a frontline clinical team, in this case, challenges arose when resources within the team were reduced by external events. Gaps in data collection were resolved by recruiting a research nurse. This study reinforces previous findings regarding the difficulty of undertaking evaluation of complex interventions, and provides recommendations for the introduction and evaluation of complex interventions within clinical settings, such as prioritisation of research within the clinical remit.

**Trial registration:**

Current Controlled Trials ISRCTN68856013, registered Nov 2010.

## Background

Given the forecast increasing prevalence of chronic obstructive pulmonary disease (COPD), current models of care provision are unsustainable and must adapt to embrace prevention and self-management [1]. This potentially requires individuals diagnosed with COPD to be supported to manage the disease at home, thereby avoiding hospital admission and reducing healthcare costs [2].

COPD is characterised by progressive worsening of lung capacity. Patients with advanced COPD typically experience impaired physical, emotional, and social functioning which results in poor quality of life [3]. In the UK, COPD is the fifth largest cause of mortality and the second largest cause of emergency admissions to hospital [4]. COPD costs the National Health Service (NHS) over £800 million per annum [5].

Remote monitoring of patients’ physiology and symptoms using Telehealth is considered to be highly appropriate for people with COPD, a condition which is associated with frequent hospital admissions and high levels of disability and depression [6, 7]. It involves the remote exchange of patient data (e.g., vital signs) between a patient and clinician which can be used to identify potential deterioration, prevent avoidable hospital admissions, and help improve an individual’s quality of life [8]. Telehealth also has the potential to help patients improve their ability to self-manage their condition, e.g., through patient education [7].

A recent Cochrane review [7] demonstrated the potential for Telehealth in reducing hospital admissions and increasing quality of life; however, in the identified studies, Telehealth was usually delivered as part of a more complex package of care and, thus, it was difficult to separate the effect of the technology from other aspects of the service. The recently reported results of a large scale UK trial of Telehealth also demonstrated that use of this technology within services has the potential to reduce mortality and emergency admission rates [9]; however, the results were not definitive in that the same study failed to demonstrate cost-effectiveness. Nevertheless, implementation of Telehealth remains a policy priority in the UK [10] and internationally, for example, through the Veterans’ Association in the US [11] and across Canada [12]. Despite the cited benefits of Telehealth [13], research has illustrated that patients and front line clinicians may not be receptive to the intervention, with the extent of staff training and support that is necessary to embed such technology into routine practice being emphasised [14].

This paper reports results from the pilot stage of a pragmatic randomised controlled trial (RCT) of a Telehealth-supported service and a standard service pathway provided through one hospital discharge service for people with COPD within one primary care trust (PCT) in the UK. Findings are reported in accordance with the Consolidated Standards of Reporting Trials (CONSORT) checklist for clinical trials [15].

### Study context

In May 2009, a PCT in the North of England introduced a dedicated discharge service for individuals with early stage COPD as defined locally^a^. It had been identified by the PCT that approximately 1,200 patients with COPD were being discharged from local acute in-patient services annually. A standard (non-Telehealth-supported) service was established to assist patients to manage their illness more effectively following hospital discharge, with the aim of decreasing readmission rates. Two COPD specialist nurses, one specialist physiotherapist, and one community matron were employed full time within the service. The COPD supported discharge service received referrals from the local NHS Acute Care Trust via two routes: i) specialist COPD nurses based on respiratory wards who were able to refer patients directly and ii) a telephone referral route allowed any relevant staff member within the hospital to refer a patient with a diagnosis of COPD, even if COPD was not the primary cause of their admission (see below for full referral criteria).

**Criteria for referral to the supported discharge service**

- ● SpO_2_ > 90% on air or pO_2_ > 7 kPa/pH 7.35–7.45
- ● Respiratory rate <25
- ● Temperature <37.8°C
- ● Systolic blood pressure 90–180 mm/Hg
- ● Pulse 50–100 BPM
- ● Orientated and alert/able to give consent
- ● Safe discharge environment
- ● Between 1 and 3 previous admissions (including the current admission) in the previous 12 months from the current date of discharge where COPD is the primary or secondary documented reason for hospitalisation

The service involved six home visits over the 8-week time frame, resulting in a conservative estimate of 8 hours and 25 minutes of time spent with each patient (including clinical administration time and cancelled appointments). It was recognised by the PCT that a service which was delivered entirely face-to-face was unsustainable in the long term, particularly given the numbers of patients presenting to acute care with COPD and in the context of an ageing population and static NHS budgets. A subsequent decision was taken by the PCT to introduce Telehealth within the discharge service. The selected Telehealth system (Doc@Home®) enables the patient to undertake daily vital signs monitoring. If monitored signs and symptoms fall outside anticipated parameters for the individual, or if the user fails to undertake monitoring activity, clinician alerts are generated so that appropriate action can be taken.

Table 1 provides an overview of the standard service and Telehealth-supported service. It was estimated that reduction of the number of home visits and inclusion of remote monitoring of patients’ vital signs could reduce the average time spent on each patient to 5 hours 30 minutes, thus better utilising staff resources and reducing costs. Telehealth equipment was to be provided to patients for 8 weeks, in accord with the overall service offer. Agreements were established between the PCT, Local Authority, and equipment provider for the installation, delivery, alert management, and de-installation of the equipment. Both the standard service and the Telehealth-supported service are free at the point of delivery for its users.

**Table 1:** **Summary of standard and Telehealth-supported services**

Timeline	Standard COPD service	Telehealth-supported COPD service
1 day – First home visit after hospital discharge	Home visit	Home visit
3 days	Home visit	Home visit
5 days	Home visit	Home visit
		Telehealth equipment installed
2 weeks	Home visit	Remote review of Telehealth parameters throughout 8 weeks
6 weeks	Home visit	Remote review of Telehealth parameters throughout 8 weeks
8 weeks	Discharge home visit	Discharge home visit
		Telehealth equipment removed

### The research programme

The overall research questions (agreed with the PCT service commissioners and managers) were as follows:

- ● Does a Telehealth-supported discharge service decrease hospitalisations compared to the standard service?
- ● Does a Telehealth-supported discharge service result in improved quality of life for people with COPD compared to the standard service and does this change over time?
- ● Does a Telehealth-supported discharge service reduce use of NHS resources compared to the standard service?

Study design was informed by the Medical Research Council guidance for evaluating complex interventions [16]. It involved a feasibility study (reported elsewhere) which investigates the practicalities of undertaking the research, e.g., flow of referrals into the discharge service and clinician/patient engagement levels. The feasibility study was followed by a pilot randomised controlled trial (RCT) to finalise study methods. The intention was to then continue to a full RCT, as described in the published protocol [17].

The specific objectives of the pilot randomised controlled trial were to i) test the trial methodology, namely recruitment, randomisation, intervention implementation, and outcome measurements; ii) estimate the sample size required for a full trial, through analysis of data on patient contact with other services, including hospital admissions; and iii) conduct a preliminary evaluation of the cost-effectiveness of the Telehealth intervention through analysis of healthcare usage, patient contact data, and quality of life data.

## Methods

The study reported in this paper involved a pragmatic two-arm pilot RCT informed by the findings from a prior feasibility study. The pilot RCT followed an ‘internal/external’ design [18] so that, if the chosen methods were consistently and rigorously applied, the pilot data might be incorporated into a full trial.

The pilot trial was conducted over 14 months. Throughout the data collection period close contact was maintained with all stakeholders via monthly project steering meetings. Records were maintained of emergent issues and identified solutions. The feasibility study identified that a target sample size of n = 60 participants, recruited over a 3 month period, would be acceptable for the pilot RCT given the number of referrals into the service and an estimated acceptance rate of 32% [17]. As shown in Table 2, potential candidates were approached by a COPD discharge team clinician during the first post-discharge home visit and were provided with study information if they expressed interest in participating. During the second visit 48 hours later, further information was provided and written consent then obtained from those who wished to participate. Random allocation to the two arms of the trial was generated through a web-based programme, accessed by the administrator for the COPD service, who generated the allocation online and informed the clinician immediately following receipt of consent. Participants received their allocated treatment pathway for 8 weeks, with a subsequent 6-month follow-up period after being discharged from the service (total trial time frame of 8 months). It was not possible to blind the involved parties due to the complex nature of the intervention and the study design.

**Table 2:** **Care pathways and integrated research activity**

	Timeline	Standard COPD service	Telehealth-supported COPD service
**Intervention**	1 day – First home visit after hospital discharge (baseline time 0)	Home visit (Trial info pack)	Home visit (Trial info pack)
	3 days	Home visit:	Home visit:
		Consent	Consent
		Baseline SGRQ (paper)	Baseline SGRQ (paper)
		Baseline EQ-5D (paper)	Letter to inform GP of trial
		GP record diaries (paper)	
		Letter to inform GP of trial	
	5 days	Home visit	Telehealth equipment installed
			Baseline EQ-5D (device)
			GP visit data (device)
	2 weeks	Home visit	Remote review of Telehealth parameters throughout 8-week intervention
	5 weeks	5-week EQ-5D (paper)	5-week EQ-5D (on device)
	6 weeks	Home visit	
	8 weeks	Discharge home visit:	Discharge home visit:
		8-week SGRQ (paper)	8-week SGRQ (paper)
		Given materials for 6-month SGRQ	Given materials for 6-month SGRQ
		Given monthly EQ-5D and GP visit diaries	Given monthly EQ-5D and GP visit diaries
			Telehealth equipment removed
**Follow-up**	8 months (6 months after discharged from service)	Measurement of outcomes (postal return):	Measurement of outcomes (postal return):
		Completion of 6-month SGRQ	Completion of 6-month SGRQ
		Completion of monthly EQ-5D	Completion of monthly EQ-5D
		Completion of monthly GP record visit diaries	Completion of monthly GP record visit diaries

EQ-5D, EuroQol 5 Dimensions; GP, General practitioner; SGRQ, St. George’s Respiratory Questionnaire.

The COPD discharge team were responsible for collecting completed self-report data during the 8-week intervention. Although it was recognised that this approach would need to be carefully monitored, clinicians were involved in data collection for a number of reasons:

- ● The nursing team were concerned that patients may become stressed if receiving multiple visitors (e.g., COPD nurse plus research nurse) whilst recovering from severe illness;
- ● The commissioning team wished to generate robust evidence but had limited funds with which to do this;
- ● One of the remits of the funding body (Collaboration for Leadership in Applied Health and Research Care for South Yorkshire) was to involve multiple partners in research, including frontline clinical staff, in order to bridge the gap between research and implementation in healthcare [19].

The clinical team underwent Good Clinical Practice (GCP) training and were carefully instructed in the requirements of the protocol.

### Inclusion/exclusion criteria

The recruitment inclusion/exclusion criteria were as follows:

### Inclusion criteria

- ● Between one and three previous admissions (including the current admission) in the previous 12 months from the date of discharge with COPD as the primary or secondary documented reason for hospitalisation;
- ● Referred to the COPD discharge service;
- ● Willing to consider using Telehealth as part of the discharge plan;
- ● Able to communicate in English and read English (a requirement of the technology);
- ● Have a telephone landline in the home (a requirement of the technology).

### Exclusion criteria

- ● Cognitive impairment to the extent that it impedes ability to participate;
- ● Other significant impairment(s) which restrict ability to participate;
- ● Existence of co-morbidities which require ongoing intervention from other community services;
- ● More than three hospital admissions for COPD within the prior 12 months;
- ● General practitioner (GP) identifies that person is unsuitable to participate (e.g., due to a mental health condition which could affect outcome measurements).

### Data collection

Table 2 provides more detail on trial pathways and data collection time points. The main planned sources of data collection were:

1. Researcher-collected data: demographic information (age and sex) were collected by the research team from routine referral records. Extracts from the Secondary Uses Service (SUS (http://www.hscic.gov.uk/sus)) database, which provides data on hospital readmissions, were provided by the (blinded) PCT statistical team to the research team for each participant for the 8 months that they participated in the trial.
2. Clinician-collected data: the St. George’s Respiratory Questionnaire (SGRQ), a validated self-measure of respiratory disease-related quality of life [20], was completed by the participant at baseline and 8 weeks, and was to be overseen and collected by the visiting clinician. SGRQ was also completed at 8 months (6 months post-intervention) and returned to the research team via post. This was supplemented by members of the clinical team calling participants to remind them to complete and send their 8-month SGRQs;
3. Device-collected data: a self-report patient-completed diary to record GP visits was recorded on paper by participants receiving the standard service and was configured to be completed on the device by those receiving Telehealth, during the 8-week intervention. During the 6-month follow-up, participants in both trial arms were asked to complete monthly paper diaries recording their GP visits. The EuroQol 5 Dimensions questionnaire (EQ-5D-3 L), a widely used measure of health outcomes standardised in a wide variety of conditions [21], was embedded into the GP diaries for those allocated to the Standard service and into the device for participants with the Telehealth-supported service.

### Outcome measures

The two co-primary outcome measures were:

1. The proportion of participants re-admitted to hospital with COPD during the 8-week intervention and 6-month follow-up, determined using SUS data on hospital readmissions; and
2. Change in self-reported health-related quality of life at baseline, 8 weeks, and 6-month follow-up through application of the SGRQ [20].

The secondary outcome measures were:

1. The proportion of patients requiring unscheduled healthcare support for the 8 week intervention period and 6-month follow-up, determined through analysis of SUS data and patient-completed diaries of GP visits; and
2. Cost-effectiveness through quality adjusted life years (QALYs) estimated from analysis of EQ-5D-3 L [21] data, GP visit data, SUS data, and the COPD discharge team’s patient contact records.

### Analysis – primary outcome measures

Analysis was conducted on an intention-to-treat basis. For the primary outcome measures, descriptive statistics are presented. No inferential statistical analyses were performed as the main objective of the study was to assess the trial methodology.

### Analysis – cost-effectiveness and cost utility

Data on hospital admissions and unscheduled healthcare support, EQ-5D scores, and COPD team patient contact records were to be accessed and analysed to provide an indication of the cost-effectiveness of each care pathway. A cost-utility analysis was carried out based on the estimated costs of Telehealth equipment, installation and de-installation of units, telemonitoring, and clinician costs. The cost of a day of COPD-related hospital admission and other secondary care services, including accident and emergency (A&E) visits, were extracted from the National Reference Costs Publication [22].

### Ethics

All necessary NHS ethical and governance approvals were obtained from the South Yorkshire Research Ethics Committee (reference 10/H130/48) and from the relevant PCT.

## Results

Throughout the trial, recruitment rate and quality of data collection were significantly impacted by an unanticipated reduction in clinical staff capacity. These factors precluded progression to a full RCT in this setting. The findings in relation to the three objectives of the pilot RCT are outlined below.

### Objective one: trial methodology

#### Recruitment

**Figure 1:**
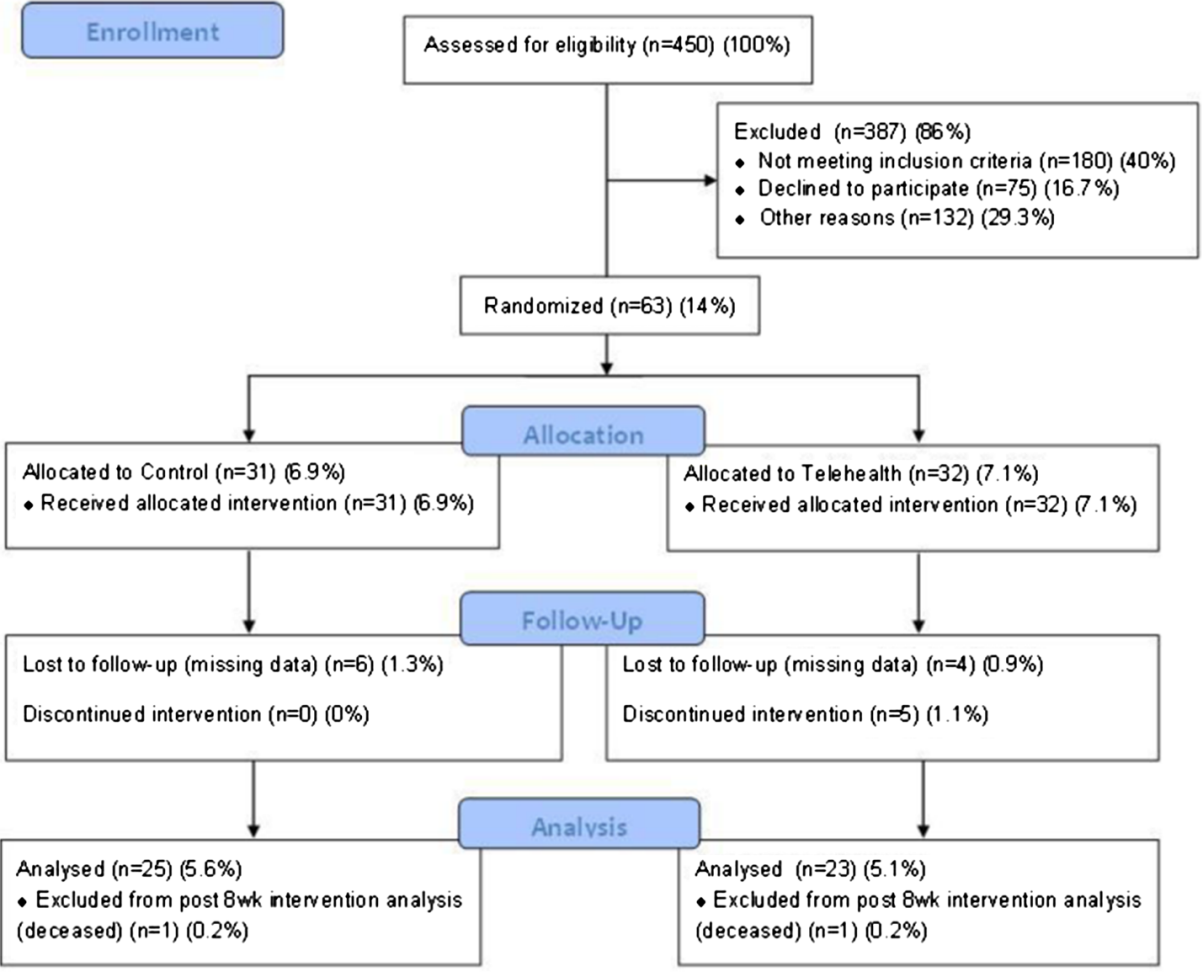
**CONSORT flow chart.**

The CONSORT [15] flow chart for the pilot trial is shown in Figure 1. Recruitment and intervention delivery took place from November 2010 to December 2011, with follow-up for 6-month post-intervention data capture continuing to June 2012. Although the recruitment target of n = 60 was achieved, this was attributed to the pilot RCT time frame being extended from 3 to 14 months due to slow recruitment to the study. During the trial time frame, 450 patients were referred to the COPD discharge service, of which 270 (60%) met the inclusion criteria. Of these, 132 could not be considered for inclusion in the study due to reasons other than declining participation, as summarised in Table 3. Some of the most common reasons were lack of clinician resources due to staff attrition that resulted in a long waiting list for admission to the service (thereby breaching the care and research protocol), immediate hospital readmission, and patient holidays/holiday periods. Of the remaining patients, 75 declined to participate and 63 (14%) agreed to enter the study and were randomised. Trial acceptance rate was 45.7% (out of 138 patients who were eligible and were not excluded for other reasons); 31 participants were randomised to the standard service and 32 participants were randomised to receive the Telehealth-supported service. The clinicians reported that refusal to participate was most often due to the person feeling too unwell.

**Table 3:** **Reasons for exclusion from trial**

Reasons (n = 132)	Number (% of eligible patients)
Backlog on telephone referral waiting list	69 (25.6%)
Not seen within adequate trial time frame	15 (5.6%)
Readmitted to hospital straightaway	11 (4.1%)
Discharged over Christmas holiday period	10 (3.7%)
Disruptions to care pathway schedule	6 (2.2%)
Unable to contact	5 (1.9%)
Patient does not believe they have COPD	4 (1.5%)
Care home resident	3 (1.1%)
Unknown clinical reason	3 (1.1%)
Going on holiday	2
Discharged elsewhere	2
Leaving area	1
Offered trial previously	1

Of the 63 randomised participants, data for 10 (15.9%) were excluded from analysis as data completion was inadequate. A further 5 participants randomised to the Telehealth-supported service were lost to follow-up (Table 4). In three instances, this was due to unexpected problems with technology connectivity despite the existence of a protocol to screen out such problems. One participant in each group died prior to completing the 6-month follow-up.

**Table 4:** **Reasons for discontinuing Telehealth intervention**

Reason	Number
No landline	2 (0.4%)
Unable to install	1 (0.2%)
Refused unit at installation stage	1 (0.2%)
Found unit difficult to use	1 (0.2%)

#### Protocol adherence and success of data collection strategy

Table 5 summarises the completeness of data collection at each time point and provides an indication of extent of adherence to the research protocol. A total of 83.3% of participants completed the intervention within the 8-week service delivery time frame.

**Table 5:** **Data completion for research outcomes**

	Standard	Telehealth	Total
**Total randomised**	**31 (100%)**	**32 (100%)**	**63 (100%)**
**Consent**			
Valid consent	25 (80.6%)	28 (87.5%)	53 (84.1%)
Missing consent	6 (19.4%)	4 (12.5%)	10 (15.9%)
**Dropped out**			
Prior to 8-week completion	0 (0.0%)	5 (15.6%)	5 (7.9%)
Prior to 8-month completion	1 (3.2%)	1 (3.1%)	2 (3.2%)
**Intervention length**			
8 weeks	20 (64.5%)	20 (62.5%)	40 (63.5%)
Less than 8 weeks	0 (0.0%)	0 (0.0%)	0 (0.0%)
More than 8 weeks	5 (16.1%)	3 (9.4%)	8 (12.7%)
**Baseline SGRQ**			
Valid	16 (51.6%)	14 (43.8%)*	30 (47.6%)
Invalid**	5 (16.1%)	3 (9.4%)	8 (12.7%)
Missing	4 (13.0%)	7 (21.9%)	11 (17.5%)
**8-week SGRQ**			
Valid	11 (35.5%)	15 (46.9%)	26 (41.3%)
Invalid	2 (6.5%)	1 (3.1%)	3 (4.8%)
Missing	12 (38.7%)	7 (21.9%)	19 (30.2%)
**6-month SGRQ**			
Valid	8 (25.8%)	5 (15.6%)	13 (20.6%)
Invalid	1 (3.2%)	1 (3.1%)	2 (3.2%)
Missing	5 (16.1%)	5 (15.6%)	10 (15.9%)
Had not reached time point by Jan12	10 (32.3%)	11 (34.4%)	21 (33.3%)
**Participants with valid baseline & 8-week SGRQ data**	9 (29.0%)	12 (37.5%)	21 (33.3%)
**Participants with valid baseline, 8-week & 6-month SGRQ data**	2 (6.5%)	2 (6.3%)	4 (6.3%)

*One participant who dropped out completed the baseline SGRQ; **Not enough completed questions to generate a valid score.

SGRQ, St. George’s Respiratory Questionnaire.

After 6 months of data collection an audit was conducted to determine adherence to data collection procedures. Significant gaps were identified which resulted in the initiation of additional procedures. The most significant change was the introduction of a research nurse in month 10 to take consent and collect trial-related data, thereby relieving the clinical team of this responsibility. Protocol adherence during the 8-week intervention increased to 100% after the research nurse was involved. As demonstrated in Table 5, there were many instances of missing or invalid SGRQ data, with 56.6%, 54.2%, and 28.3% valid completion rates for each respective time point (when excluding drop-outs and missing consent). Only 43.8% of participants had a valid SGRQ score for both baseline and 8-week time points, and this figure reduced to just 8.7% for all three time points. The SGRQ is designed to be completed by the participant overseen by a researcher or healthcare professional. Although this was agreed in the protocol, clinicians tended to leave the SGRQ with participants to complete in their own time. Clinician feedback during the pilot was that they did not feel comfortable overseeing SGRQ completion as they were wary of biasing participants’ responses when asked for advice.

The completion rate for the standard service 8-week self-report diaries (which included the EQ-5D) was similarly challenged. The standard service group EQ-5D completion rate at baseline was 72.0% with a 5 week completion rate of 44.0%, which did not allow comparison with EQ-5D data from the Telehealth-supported group (embedded within Doc@Home). The self-report diaries for the standard service group were completed in 44.0% of cases. The postal return of monthly diaries in the 6-month follow-up (which were given to both groups) did not yield meaningful data (12.5% returned).

### Objective two: healthcare usage and sample size calculation

#### Baseline characteristics

Table 6 shows age and sex distribution for the 53 consented participants. The Telehealth-supported group contained a greater proportion of males and had a slightly higher mean age. Lack of comparability between the two trial arms was a likely consequence of the small sample size.

**Table 6:** **Baseline demographic information**

Demographic		Standard	Telehealth	Total
**Sex**				
	Male, n (%)	7 (28.0%)	12 (42.9%)	19 (35.8%)
	Female, n (%)	18 (72.0%)	16 (57.1%)	34 (64.2%)
**Age (years)**				
	Mean (SD)	65.88 (9.39)	67.22 (11.60)	66.59 (10.54)
	Median (IQR)	68.00 (58.85–72.85)	70.80 (60.93–73.73)	69.60 (59.80–73.40)
	Minimum	41.2	44.2	41.2
	Maximum	78.4	90.9	90.9

#### Healthcare service usage data

Data on the frequency and length (bed days) of hospital admissions, frequency of A&E visits (which did not lead to hospital admission), and frequency and type of community healthcare service contacts (other than the COPD discharge service) were extracted for all participants (who completed the 8 week intervention) for the duration of the intervention and 6-month follow-up. Results summarised in Table 7 show that participants receiving the standard service had a lower readmission rate, fewer hospital admissions, and fewer inpatient bed days than those receiving the Telehealth-supported service. Frequency of community healthcare service contact was similar between the two groups. It was not possible to infer the number of GP visits from the available data.

**Table 7:** **Healthcare service use by participants**

	Standard	Telehealth	Total
Sample size	25	23	48
**Proportion with hospital admissions**			
Number	4	8	12
Percentage	16.0%	34.8%	25.0%
**Number of hospital admissions**			
Total	7	16	23
Mean	0.28	0.70	0.48
Maximum	3	5	5
**Total number of hospital days**			
Total	21	129	150
Mean	0.84	5.61	3.13
Maximum	14	43	43
**Proportion with A&E visits**			
Number	4	1	5
Percentage	16.0%	4.3%	10.4%
**Number of A&E visits**			
Total	5	1	6
Mean	0.20	0.04	0.13
Maximum	2	1	2
**Proportion with community nurse contacts**			
Number	4	5	9
Percentage	16.0%	21.7%	18.8%
**Number of community nurse contacts**			
Total	8	13	21
Mean	0.32	0.57	0.44
Maximum	3	4	4
**Mortality rate**			
Number	1	1	2
Percentage	4.0%	4.3%	4.2%

#### Sample size calculation

It was not possible to use the pilot RCT data to generate a sample size calculation due to incomplete data collection. Therefore, the calculation was conducted using estimates of readmission rates, clinically meaningful effect sizes based on the literature, and clinical expertise. A 10 to 20% relative reduction in hospital readmission rate was deemed to be clinically meaningful. Table 8 shows the sample sizes required to detect reductions in this range, given 90% power to detect significant differences at a *P* value of 0.05. If we take an intermediate value, with a 15% relative reduction in hospital admissions from 34% to 29%, 1,517 patients per arm (n = 3,034 total) would be required for a full RCT.

**Table 8:** **Required per arm sample size at** ***P*** **= 0.05 and 0.9 power**

Relative reduction	Absolute reduction in number of admissions per 100 discharges	Sample size per arm
10%	3	4,240
15%	5	1,517
20%	7	768

### Objective three: quality of life and preliminary evaluation of cost-effectiveness

SGRQ data analysis shows that both groups reported an increase in disease-related quality of life (decrease in SGRQ score) between baseline and 8 weeks (Table 9). However, this increase was larger in the standard service group.

**Table 9:** **St George’s Respiratory Questionnaire (SGRQ) analysis for participants with valid baseline and 8-week SGRQ scores**

Time point	Metric	Standard (n = 9)	Telehealth (n = 12)
	Mean (SD)	65.34 (13.68)*	69.63 (16.58)
Baseline (n = 21)	Median (IQR)	67.24 (52.97–77.73)	73.32 (55.16–83.85)
	Min – Max (Range)	44.77–82.25 (37.48)	35.88–88.09 (52.21)
	Mean (SD)	59.18 (13.21)	67.47 (15.70)
8-week (n = 21)	Median (IQR)	62.14 (44.52–67.98)	69.99 (55.82–76.83)
	Min – Max (Range)	42.71–81.66 (38.95)	37.18–87.88 (50.70)

*The SGRQ is scored from 0 (best possible health status) to 100 (worst possible health status).

Due to gaps in data collection the analysis method was adjusted so that EQ-5D scores were calculated from participants’ SGRQ scores using a mapping formula produced in a previous study in patients with COPD [23]. This estimates EQ-5D score as a function of SGRQ total score and sex. Quality adjusted life years (QALYs) were calculated from the EQ-5D scores using the trapezium rule. Missing data were imputed using the last observation carried forward method. Costs and QALYs were calculated for each group, and then used to plot data on the cost-effectiveness plane and to produce associated cost-effectiveness acceptability curves. A value of £20,000 per QALY was used to determine the probability that the intervention is cost-effective under current funding conditions.

The primary analysis was based on all NHS costs and was performed using estimates of unit costs and estimates of resource use. There was a higher mean total cost in the Telehealth-supported group (£1,750 vs. £580 for the standard service). Comparison with the mean cost difference showed an incremental cost per QALY gained of £68,811 (Table 10).

**Table 10:** **NHS cost and QALYs over 6 months**

Item	Standard mean (n = 25)	Telehealth mean (n = 28)	Mean difference
Total costs	£580	£1,749.8	£1,169.8
Quality adjusted life years gained	0.20	0.217	0.017
Incremental cost-effectiveness ratio			£68,811 per QALY

A secondary sensitivity analysis was carried out based on just the costs relating to community care, i.e., Telehealth and nursing contacts. The rationale for this was that hospitalisations had a disproportionate effect on the results, and being so rare in a pilot study, their mean effect was possibly largely due to chance.

The results of the secondary analysis, using only the costs of community care, showed that there is a 71.4% chance that the Telehealth-supported services are cost-effective given the willingness to pay of £2,041 per QALY gained (Table 11).

**Table 11:** **Community care costs and QALYs over 6 months**

Item	Standard mean (n = 25)	Telehealth mean (n = 28)	Mean difference
Total costs	£348.3	£383	£34.7
Quality adjusted life years gained	0.20	0.217	0.017
Incremental cost-effectiveness ratio			£2,041 per QALY

To summarise, when considering community care costs only, and estimated equipment costs of £455 over 5 years with a fairly low utilisation rate (three users per year), the Telehealth intervention is perceived to be cost-effective. However, when hospital admissions data are included in the analysis then the Telehealth intervention is not deemed to be cost-effective.

## Discussion

In this paper we describe a pilot randomised controlled trial of a Telehealth intervention for COPD. The results showed that it would not be feasible to continue the pilot trial to a full RCT. In conducting this research we have identified issues of critical importance for any subsequent study of this complex intervention, including the involvement of clinicians in recruitment and in the research process. The service commissioners’ expectation was that Telehealth-supported services might be clinically and cost-effective for people receiving time-limited support following hospital discharge. Although the pilot trial has not been able to give a robust indication of clinical or cost benefit due to its small sample size and incomplete data collection, it has achieved its objective of demonstrating the practicalities of answering these questions with a full scale RCT in this particular setting.

Compared to studies recruiting participants at multiple sites and working with several clinical teams, it could be presumed that working with one clinical team with high volume referral rates in one community would simplify the research process. The study was conducted within an existing clinical service, thus bringing increased external validity through its pragmatic design [24] and the ability to directly inform one region’s commissioning choices with regard to Telehealth. However, the process of undertaking this study reinforces findings from larger-scale studies (e.g., Hendy et al. [25]) regarding the difficulty of evaluating novel, complex interventions such as Telehealth, especially when trying to assess the utility of the intervention within a clinical service alongside undertaking rigorous research.

The study was dependent upon one small clinical team working against a backdrop of NHS restructuring. Even though initial difficulties with obtaining staff ‘buy in’ were identified and appeared to be resolved during the feasibility study, incremental demands created by the research combined with the loss of a key champion for the trial amongst the front line staff had a deleterious effect over time. Further staff attrition resulted in an eventual total loss of 60% of staff capacity within the frontline clinical team. Thus, the main challenge of involving frontline clinicians in the research and in data collection was that, understandably, clinical priorities always came first, and when resources were stretched there was little room for the rigorous research processes which were required for the pilot trial. PCT budgetary restrictions prevented return of the COPD team to its initial capacity. The combined effect of reduced staff capacity and some non-compliance with trial procedures (both staff and participants) resulted in incomplete data collection and slow recruitment to the trial. This also shows that GCP training of clinical staff is not enough to ensure adherence to trial procedures, thus research processes and data collection need to be rigorously monitored throughout the trial.

Despite the successful introduction of a Research Nurse in month 10 to consent participants and collect trial data, organisational changes within the PCT would not have allowed continuation of the pilot trial to a full RCT even if the limitations of the methodology were resolved. One example of an organisational change which impacted the project was the national reorganisation of the NHS, meaning that PCTs were disbanded in March 2013 and replaced by Clinical Commissioning Groups.

The consequences of both care pathways being relatively new within what was a recently introduced service cannot be underestimated. Fidelity to the pathways was difficult to achieve at the outset and was readily compromised. The waiting list for admission to the discharge service, which began to build in month 4 of the pilot trial due to the unanticipated 60% reduction in staff capacity, is a clear example of how the care pathways were changed due to service imperatives which were out of the control of the research team.

There were issues regarding clinician commitment to Telehealth and the work-based support they needed to deliver this new intervention efficiently and appropriately. The importance of training and on-going support to deliver Telehealth is now recognised [14], but was not available to the clinicians who participated in this study with the consequence that adherence easily eroded and confidence waned. Problems with device connectivity for some of the participants randomised to receive Telehealth despite application of the study’s inclusion/exclusion criteria reinforced the lack of clinician confidence.

Feedback from the feasibility study indicated that those receiving the clinician-delivered ‘standard service’ valued this service and benefitted from it, and that both groups expressed a preference for personalised face-to-face service. The question this poses is whether those receiving Telehealth were disadvantaged in this instance compared with those in receipt of a specialist face-to-face service, which was highly tailored to patient need but unlikely to be sustainable in the longer term. This preference for face-to-face care may be more influential than the perceived reassurance of daily monitoring provided through using Telehealth. Additionally, questions were raised by the study regarding which patients might gain most benefit from Telehealth. Study participants were recruited at the point of hospital discharge and it became evident that receptiveness to using Telehealth could be severely compromised by their illness. It is possible that asking a patient to use a piece of technology at this point, even one which is simple to use, could be a daunting commitment to take on in addition to recovering from their exacerbation. There are unanswered questions regarding when in the overall care pathway from acute to primary care can Telehealth technology be most effective and for how long, taking into account exacerbation severity. There are also questions around the representativeness of the sample, and when in the COPD disease pathway deployment of Telehealth would be of most benefit. Forty percent of patients referred to the COPD service failed to qualify for the trial on one or more of the eligibility criteria, indicating that trial participants may not have been fully representative of the COPD population as a whole and that our trial design may not have been as ‘pragmatic’ as had been intended. The definition of between one and three previous hospital admissions, which formed a key component of the eligibility criteria, was based on the local definition of early stage COPD. However, it could be argued that a person with three admissions in the previous 12 months may have more advanced disease compared to someone with one or no admissions. To summarise, commissioners and clinicians require greater guidance for deployment.

By definition, complex interventions are difficult to define, standardise, and measure, and enthusiasm to undertake research can underestimate this. The results obtained from this study question the viability of involving front line clinical staff in data collection for robust research evaluation. Whilst it is true that clinicians do not need to be involved in data collection, the observations of Bird et al. [26] provide further support regarding the importance of context and culture when conducting trials, yet this is not evidenced through on-going commissioning of trials of complex interventions.

Questions remain regarding how to most appropriately conduct local evaluations to inform commissioning decisions. Leykum et al. [27] suggest integration of participatory action research and randomised controlled trial methods to ensure that a complex intervention is adequately embedded within the setting. Arguably, this did occur within this study through the extensive involvement of commissioners and clinicians in study set up, design, and progress, and through regular meetings during the entire programme. As demonstrated by Hendy et al. [25], the drive to demonstrate population-based benefit through evaluation of complex interventions does not necessarily equate with the demands of implementing a complex intervention *in situ*.

## Conclusions

We were able to complete an informative pilot RCT, despite service reconfiguration and slow recruitment rates. However, ultimately, these factors precluded progression to a full RCT in this setting. On the basis of our experience in conducting this pilot study, we are able to recommend that a definitive trial should be multi-centre and aim to include 1,517 participants in each arm. Consent, randomisation, and data collection (in accordance with the protocol) should be supported by dedicated research staff rather than by clinicians. However, the study also raises a dilemma – there are indications that patients prefer face-to-face contact even when offered daily but remote interaction through Telehealth, yet this model of care delivery may not be sustainable in the current climate.

## Endnote

^a^Early stage COPD was defined as “Between one and three previous admissions (including the current admission) in the previous 12 months from the current date of discharge where COPD is the primary or secondary documented reason for hospitalisation”.

## Abbreviations

A&E: Accident and emergency
CONSORT: Consolidated standards of reporting trials
COPD: Chronic obstructive pulmonary disease
EQ-5D: EuroQol 5 Dimensions
GCP: Good clinical practice
GP: General practitioner
NHS: National Health Service
PCT: Primary care trust
QALY: Quality adjusted life year
RCT: Randomised Controlled Trial
SGRQ: St. George’s Respiratory Questionnaire
SUS: Secondary Uses Service.
